# How Can We Do Better? Learning From 617 Pediatric Patients With Airway Foreign Bodies Over a 2-Year Period in an Asian Population

**DOI:** 10.3389/fped.2020.00578

**Published:** 2020-09-10

**Authors:** Ying-Qin Gao, Jian Li Tan, Mei-Lan Wang, Jing Ma, Jia Xi Guo, Ken Lin, Jing-Juan Wei, De-Yun Wang, Tie-Song Zhang

**Affiliations:** ^1^Department of Otolaryngology, Head and Neck Surgery, Kunming Children's Hospital, Kunming, China; ^2^Department of Otorhinolaryngology, Tan Tock Seng Hospital, Singapore, Singapore; ^3^Department of Otolaryngology, Head and Neck Surgery, Kunming Children's Hospital, Kunming Medical University, Kunming, China; ^4^Department of Otolaryngology, Yong Loo Lin School of Medicine, National University of Singapore, Singapore, Singapore

**Keywords:** pediatric, foreign body, chest radiography, bronchoscopy, airway, medical management

## Abstract

**Background:** Foreign body (FB) in the pediatric airway is a prevailing and crucial emergency with presenting symptoms often overlapping with other common pediatric conditions. There are limited number of large cohort studies in an Asian population which demonstrate the diversity of symptoms, investigations which will aid in obtaining the diagnosis, and management. Using this large cohort, we aim to evaluate the type and location, clinical presentations and outcomes of medical management related to pediatric airway FB in an Asian society.

**Methods:** This is a retrospective study of all airway FB treated in Kunming Children's Hospital, China from February 2016 to June 2019. Six hundred and thirty-two clinical and operative records of all airway FB were retrieved and reviewed from the hospital's central electronic medical records. A total of 617 patients were included in our study.

**Results:** The age ranged from 4 months to 12 years (mean = 1.74 years). The duration of symptoms ranged from 1 h to 605 days, with the diagnosis established at an average 9.16 days. Almost all had multiple symptoms, most commonly cough (98.5%) followed by noisy breathing (98.2%). Majority of the FBs (95.5%) were organic and the rest inorganic. Of the organic FBs, peanut was the most common (31.6%), followed by walnut (13.3%) and sunflower seeds (9.2%). Comparatively, 80.8% of the organic FBs were retrieved incomplete while 85.7% of the inorganic FBs were completely intact. Multiple FBs were noted in 43.3% of the patients, with 2.4% of them in different locations.

**Conclusions:** Airway FB can be easily missed with resultant delay in diagnosis. In an Asian population, walnut and sunflower/pumpkin seeds feature more prominently compared to Western populations. Sunflower seed FBs tend to present earlier and are found intact in the trachea. Rigid bronchoscopy is the most common technique used to remove such FBs and pulmonary-related complications post-operatively, though rare, are the most common adverse outcomes. Preventive strategies targeting the appropriate age group and this type of FB may be useful in an Asian population.

## Introduction

Foreign body (FB) in the pediatric airway is a prevailing and crucial emergency that every practitioner involved in pediatric care should be well-versed in. Ingestion or aspiration of FB was responsible for thousands of emergency department visits and more than 150 deaths each year in the United States (1, 2). It is estimated to cost $41 million annually in inpatient costs (3). This condition often provides a diagnostic dilemma as many of the presenting symptoms overlap with other common pediatric conditions. Airway FB carries a mortality rate of up to 2.75% and should not be treated lightly (4). Despite existing legislation to regulate toys and other inorganic items which may be a choking hazard, airway FB is still the leading cause of accidental infantile deaths and the 4th most common cause of death among preschool aged children (5).

This is one of the largest Asian cohort studies regarding this topic, as prospective studies are not practical or feasible. Using this large cohort (*n* = 617), we aim to evaluate our hypothesis that pediatric airway FBs in an Asian society have differences in type and location of FB, as well as outcomes of management. We intend to highlight the cultural differences in an Asian society which warrant a different approach toward preventive strategies in pediatric airway FB. We seek to highlight to non-otorhinolaryngologists regarding the myriad of presenting symptoms, differential diagnoses, investigations which will aid in obtaining the diagnosis, as well as management. With this information, we hope to avoid unwarranted urgency or delayed diagnosis.

## Materials and Methods

**Figure 1:**
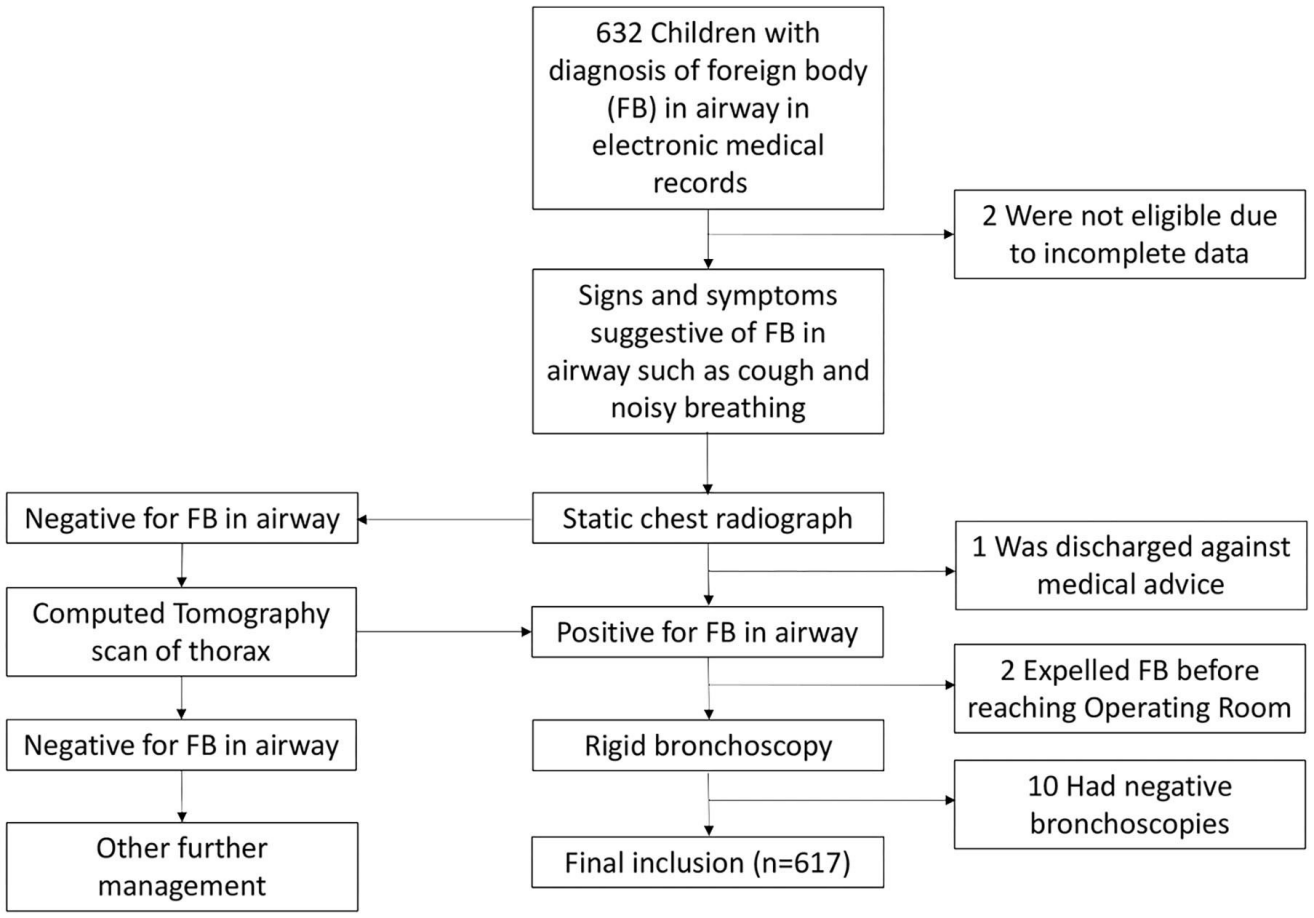
Flowchart showing methods of patient collection and management in this study. The clinical and operative records of 632 children with airway foreign body (FB) treated in Kunming Children's Hospital, China from February 2016 to June 2019 were retrieved and reviewed. The common signs and symptoms suggestive of FB in airway were analyzed, together with the standard of diagnosis and management of the patients.

This retrospective study was performed at Kunming Children's Hospital, the largest tertiary pediatric hospital with 900 patient beds in Yunnan Province (estimated population of 46 million), People's Republic of China. Ethical review and approval were not required for the retrospective study on human participants in accordance with the local legislation and institutional requirements. Written informed consent from the participants was not required to participate in this study in accordance with the national legislation and the institutional requirements. Clinical records of all airway FB treated in this hospital from February 2016 to June 2019 were retrieved and reviewed from the hospital's central electronic medical records. A total of 632 clinical records were reviewed, with inclusion of 617 patients in our study. Fifteen were excluded due to incomplete data, patients discharged against medical advice or expulsion of the FB before reaching the Operating Room. Figure 1 shows the methods of patient collection in this study.

Data collected included demographic data and the history of presenting complain taken from the patient and accompanying parent; these included details such as history of FB ingestion, type and duration of presenting symptoms. Pre-operative investigations such as chest X-ray and/or Computed Tomography (CT) scan of the thorax were also retrieved. Operative procedure and operative findings such as location, number, and type of FB as well as presence of granulation tissue were retrieved from the operating records. Lastly, the need for Pediatric Intensive Care Unit (PICU) care, reason for PICU admission, duration of hospitalization, complications, and mortality were also reviewed.

All data were analyzed using SPSS version 23.0 (SPSS Inc., USA). Normality of all continuous variables were examined using the Shapiro–Wilk test. The Kruskal–Wallis test was used to compare the distribution of age and duration of symptoms between FBs in different location. Chi-square test was performed to compare (1) the percentage of PICU admission between FBs in different location, (2) integrity of different type of FB, and (3) distribution of type of FBs in different location.

## Results

The age of presentation ranged from 4 months to 12 years (mean age of 1.74 ± 1.61 years) (Table 1). Amongst our 617 patients, 64.3% (*n* = 397) of the patients were male and 35.7% (*n* = 220) were female. The duration of symptoms ranged from 1 h to 605 days (mean duration of 9.16 ± 30.93 days). More commonly, patients were diagnosed slightly after a week (mean 9.16 ± 30.93 days). Six patients (1%) presented with symptoms >3 months, 21 patients (3.4%) >2 months, and 45 patients (7.3%) >1 month. There was a 5-year old girl who presented with airway symptoms (cough and wheeze) for 605 days who was being treated as bronchitis by the local physicians. She was admitted to PICU and intubated due to exacerbation of dyspneic for 5 days. A tiny plastic ring was taken from her trachea with the formation of granulation.

**Table 1:** Age, duration of symptoms, and Pediatric Intensive Care Unit (PICU) admission by location of foreign body.

**Location of FB**	***N* (%)**	**Age (years) mean ± SD**	**Duration of symptoms (days) mean ± SD**	**PICU admission *N* (%)**	
				**No**	**Yes**
Right main bronchus	267 (44.7)	1.69 ± 1.47	9.84 ± 23.59	252 (94.4)	15 (5.6)
Left main bronchus	222 (37.1)	1.70 ± 1.68	8.47 ± 18.12	206 (93.8)	16 (7.2)
Trachea	95 (15.9)	2.13 ± 1.90	0.88 ± 62.15	87 (91.6)	8 (8.4)
		*p* = 0.072	*p* < 0.01^*^	*p* = 0.59	

*Kruskal–wallis test was used to compare the means between the 3 groups in each column*.

* *Sub-analysis showed significant difference between trachea and right or left main bronchus, but there was no significant difference between right and left main bronchi*.

Patients with airway FB presented with more than one symptom. 99.7% had more than one symptom, most commonly cough (98.5%) and noisy breathing (98.2%). Other less common symptoms included respiratory distress (14%), fever (3.4%), and cyanosis (3.2%).

With regards to the FB, 95.5 % (*n* = 589) were organic and 4.5% (*n* = 28) were inorganic. Of the organic FBs, peanut was the most common (31.6%), followed by walnut (13.3%) and sunflower seeds (9.2%). Of the organic FBs, 80.8% were retrieved incomplete, compared to majority (85.7%) of the inorganic FBs which were completely intact; this was statistically significant (*p* < 0.05). Inorganic FB also tended to be single (89.3%) when compared to organic FB (55.2%). Pen caps account for 35.7% (*n* = 10) of the inorganic FBs which is the most common. Multiple FBs were noted in 43.3% of the patients (*n* = 268), with 2.4% of them (*n* = 15) in different locations. Of the multiple FBs, the highest number was 9 pieces of walnut. Significantly, 3.7% of these patients also had FBs on the contralateral side. Sunflower and pumpkin seeds were more likely to be intact (58.1%) as compared to peanuts (0.4%). Granulation tissue was associated with the FB in 6.8% of our patients. While we did not find any statistically significant correlation with duration, symptoms or PICU stay, all cases associated with granulation tissue were secondary to organic FB, without preference for any particular organic FB.

As expected based on the anatomy of the bronchi, 42.5% of the FBs were in the right main bronchus and 38.2% in the left main bronchus. The rest were found in the trachea (16.4%) and larynx (2.4%). Six hundred and sixteen patients (99.8%) underwent rigid bronchoscopy while 4 patients (0.6%) underwent flexible bronchoscopy. Three patients underwent both rigid and flexible bronchoscopy.

As there were only 14 cases of laryngeal FBs, we only excluded these cases when we analyzed data regarding the location of FB to preventing skewing of results. We also further analyzed the distribution of the type of FBs by location; peanut was less commonly found in the trachea whereas bone and sunflower seeds are more likely to be found in the trachea (*p* < 0.01). This is presented in Table 2.

**Table 2:** Distribution of type of foreign bodies (FBs) by location.

**Type of FB**	**Location of FBs. *N* (%)**		
	**Right main bronchus**	**Left main bronchus**	**Trachea**
Peanut	94 (49.7)	84 (44.4)	**11 (5.8)**
Corn	4 (44.4)	2 (22.2)	3 (33.3)
Pea	7 (38.9)	7 (38.9)	4 (22.2)
Bone	5 (25.0)	7 (30.0)	8 (40.0)
Other organic FBs	22 (45.8)	18 (37.5)	8 (16.7)
Inorganic FBs	11 (44.0)	9 (36.0)	5 (20.0)
Sunflower seed	16 (28.6)	13 (23.2)	**27 (48.2)**
Broad bean	8 (34.8)	12 (52.2)	3 (13.0)
Chestnut	20 (60.6)	10 (30.3)	3 (9.1)
Pumpkin seed	17 (35.4)	15 (31.3)	**16 (33.3)**
Walnut	45 (57.7)	30 (38.5)	3 (3.8)
Nut	12 (46.2)	13 (50.0)	1 (3.8)
Soybean	6 (54.5)	2 (18.2)	3 (27.3)

*Bold = p < 0.01*.

Forty-eight (7.8%) patients required post-operative PICU care. The average duration of hospitalization for these patients was 9.65 ± 4.28 days. This was attributed mainly to respiratory issues (91.7%); most commonly due to difficult extubation and pneumonia. The mortality rate in our study was 0.2% (*n* = 1). This boy who was <2 years old, presented with cyanosis and altered mental status. He asphyxiated and went into cardiopulmonary arrest. Despite resuscitation and removal of the FB (grape lodged in the trachea), the cerebral damage was irreversible and the child passed away eventually. Out of the 48 patients who require PICU stay, 27.1% of them were below the age of 12 months.

## Discussion

In our study, 94.8% of the patients are <5 years old and 64.3% of them are male. This is consistent with other studies which identified risk factors for airway FB to be age <5 years old, male gender and private insurance status (2, 3, 6, 7). Children of that age have underdeveloped swallowing due to lack of full posterior dentition and mature neuromuscular mechanisms. They also have narrower airways which may not permit expulsion of foreign objects by cough or the Heimlich maneuver. Additionally, the developing child will often place items into their mouths while exploring their environment and may not be attentive to the task of eating (8). In the catchment area around Kunming Children's Hospital, most children attend preschool between the ages of 3 to 6. As such, education and preventive efforts via coordinated preschool education targeted at these preschool children and their parents may benefit significantly. As healthcare in this population is subsidized by the government, insurance status is unlikely to play a role as compared to other countries.

Interestingly, the longest duration of symptoms (according to history taken from the parents) in our study was 605 days prior to referral to a tertiary hospital, suggesting that in rare cases, airway FB can be present without causing symptoms severe enough to seek medical attention. Forty-five (7.3%) of our patients presented with more than 1 month of symptoms. More commonly, patients are diagnosed slightly after a week (mean 9.16 ± 30.93 days). We attribute this delay to the overlapping symptoms with other common conditions for which the children are likely treated for, prior to consideration of pediatric airway FB as a possible differential diagnosis. This reflects the challenge in the prompt diagnosis of pediatric airway FB. Further education of non-otorhinolaryngologists such as General Practitioners and Pediatricians may increase awareness and heighten the index of suspicion for airway FB. However, these physicians should also realize that pediatric airway FB can be present for a long time and yet do not produce very significant symptoms. This will also prevent unwarranted anxiety to the parents or care-givers who may demonstrate self-blame behavior.

We also found that FB in the trachea tend to have a shorter duration of symptoms compared to those in the bronchus (Table 1). This could be due to the severity of symptoms leading to earlier presentation in patients with FB in the trachea. Age and PICU admission did not seem to have a correlation with the location of FB.

When we reviewed the presenting symptoms of the patients with airway FB, we found that 99.7% of them have more than one symptom of which cough and noisy breathing are the most common symptoms. In the institution where this study was conducted, if the history and physical examination is suggestive of an airway FB, a static chest radiograph (CXR) is performed (9). If these investigations are positive, the patient undergoes a rigid bronchoscopy. If they are negative, CT scan of the thorax is performed to increase the pick-up rate (10, 11).

Pediatric airway FB has always been a tricky diagnosis due to a myriad of factors (12). Physicians such as general practitioners or pediatricians are frequently the first line of consult for a pediatric patient with potential airway FB. Very often, the history may not be clear and the symptoms overlap with common pediatric diseases. However, should there be a myriad of symptoms such as above, the physician should always consider airway FB especially if symptoms are recurrent or non-resolving. With a vague history at best and subtle (sometimes absent) physical and CXR abnormalities, the diagnosis of FB airway can be tricky. This is also well-described in the literature (13, 14). Some authors have proposed a clinical algorithm scoring system to aid in diagnosis of suspected FB aspiration in children. One such system proposed by Janahi et al. (15) was based on 300 patients using 5 predictors: witnessed choking, noisy breathing/stridor/dysphonia, new onset/recurrent/persistent wheeze, unilateral reduced air entry, and abnormal CXR. These 5 predictors were assigned a weighted risk score of 1, 1, 2, 1, and 2, respectively. Patients with a score of 5 or more had bronchoscopy-proven FB in the airway in 72% of cases. This may be a useful algorithm for clinicians to incorporate into their practice. It is also useful for non-otorhinolaryngologists to know that bronchoscopy has an overall low incidence of 30-day adverse events (16).

Our results highlighted the importance of completing the bronchoscopy to check the distal as well as the contralateral airway after retrieving the first FB that is seen. This can be the pitfall of even the most competent surgeon. As such, the authors recommend to always perform a full bronchoscopy to ensure that there are no additional FBs as well as to check for any granulation or injury to the tracheobronchial tree. Rarely, structural airway abnormalities can be picked up as well (17).

All cases associated with granulation tissue were secondary to organic FB but no correlation was found between the different types of organic FBs or with the duration of PICU stay. It has been proposed that organic FBs may cause more granulation formation due to mechanical and chemical irritation. Chemical alkaloid effects can occur due to the release of biologically active alkaloids present in certain nuts (18). This finding suggests that nuts may incite a more inflammatory reaction and be possibly associated with more complications. Granulation tissue or increased inflammatory reaction around the FB may increase the risk of airway stenosis. In the literature, there is few data on delayed airway stenosis as a complication of airway FB. Frequently, patients with airway FB do not undergo another bronchoscopy after a successful removal of the FB. However, there have been reports of surveillance bronchoscopy picking up delayed stenosis; early therapeutic interventions such as balloon bronchoplasty can help to restore airway patency and prevent lung damage (19). As such, there may be a role in including a surveillance bronchoscopy into our protocol for patient with significant granulation or inflammation around the impacted FB. Majority of the patients (93.8%) who required PICU care post-operatively were associated with organic FB suggesting that organic FBs and their resultant inflammatory effects may occlude the airways more significantly resulting in pulmonary-related complications (20). However, this data may be skewed because of the significantly smaller number of inorganic FB.

Organic FBs were a lot more common than inorganic FBs. Hotdogs have been associated with fatal choking incidents due to their oval and compressive nature (21). However, we do not have a single case of airway FB due to hotdogs in our series due to the cultural differences in diet. Sink et al. looked at their series of 83 cases and reported that more than half of their FB were fragments of seeds and nuts and peanuts were the most common (22). Our cohort was similar but we also noted a high incidence of walnut, sunflower, and pumpkin seeds. This finding was interesting and to our knowledge, not mentioned in the literature previously. This is likely due to cultural and dietary differences found in an Asian population. It is pertinent to note that the average sizes of sunflower and pumpkin seeds are smaller than peanuts which may explain why they are found intact more often than peanuts. Public education regarding the types of seeds and nuts that carry a high risk of airway FB is crucial. Despite existing legislation to regulate toys and other inorganic items which may be a choking hazard, efforts should also be targeted toward organic FBs which still form the majority of our airway FBs. In an Asian society, perhaps emphasis should be placed on sunflower and pumpkin seeds.

From the data that was presented in Tables 1, 2, FBs in the trachea are more likely to present earlier as well-compared to other types of FBs (23). The flat and elliptical shape of sunflower seeds poses a unique problem in the Asian society. They are smaller and tend to be inhaled intact to lodge in the trachea where FBs present earlier as compared to other locations as described earlier.

The most common inorganic FB in our series is pen caps which is easily available to a toddler in a domestic setting. While legislative regulations put in place in many countries to target toys have had relative success, consideration should be given to FBs such as nuts and pen caps. As these are ubiquitous objects, preventative strategies targeted at cultivating awareness and appropriate labeling of such items may be more useful (21).

Most FBs were in the right main bronchus which was expected due to less acute angle of the right main bronchus compared to the left. A study using information from the Kids' Inpatient Database (KID) (24) showed that lower airway FB increases the need for mechanical ventilation. Anecdotally, the authors think this may be due to higher likelihood of mucus plugging or granulation formation resulting in pneumonia or secondary lung processes.

Lastly, we examined the morbidity and mortality associated with airway FB in our series. This was similar to a cross-sectional analysis of a US National database by Tan et al. (16) which showed an adverse outcome rate of bronchoscopy and removal of tracheobronchial foreign bodies in children to be 4%, out of which most were pulmonary-related complications.

Sheehan et al. (17) showed that patients <12 months that had a suspected airway FB had a very low rate of positive bronchoscopy. However, we would like to highlight that this group of patients are more prone to complications given their young age. The authors recommend to have a low threshold to suspect airway FB in this group of patients. In addition, 15 out of 16 patients in the above study have structural airway abnormalities which could also be detected via a bronchoscopy making it both diagnostic and possibly therapeutic in the event of an airway FB.

Our mortality rate (0.2%) was lower than the mortality rate of 1.8% reported by Kim et al. based on the Nationwide Inpatient Sample database (3) as well as 2.5% by Johnson et al. using the Kids' Inpatient Database (24). Fidowski et al. (25) analyzed 12,979 cases of airway foreign bodies in children through a systematic review and determined the mortality rate of bronchoscopy to be 0.42% which was closer to our study.

In our study, patients with expelled or negative FBs were excluded. There was a total of 10 bronchoscopies that were negative. Some of the patients with negative FBs may be due to very small FBs which may have been removed during suctioning of the mucus. If patients expelled the FBs and were asymptomatic thereafter, a bronchoscopy was not performed. Documentation of granulation tissue was also up to the discretion of the clinicians, it could be under-reported as only severe cases were documented. The lack of correlation between granulation tissue and duration of symptoms or need for PICU stay may be due to the small number of cases being documented. Being a retrospective study, we also do not have prospective data regarding the long-term complications of airway FB such as airway stenosis.

## Conclusion

In this study, we present one of the largest Asian cohort on pediatric airway FB. Airway FB can be easily missed or delayed in diagnosis. Non-otorhinolaryngologists should have a high index of suspicion but yet understand that airway FB can be present for a long time without causing significant symptoms. In an Asian population, walnut, and sunflower/pumpkin seeds feature more prominently compared to Western populations. Sunflower seed FBs tend to present earlier as they are found intact in the trachea. Rigid bronchoscopy is the most common technique used to remove such FBs and pulmonary-related complications post-operatively, though rare, are the most common adverse outcomes. Preventive strategies targeting the appropriate age group and this type of FB may be useful in an Asian population.

## Data Availability Statement

All datasets generated for this study are included in the article/Supplementary Material.

## Ethics Statement

Ethical review and approval was not required for the study on human participants in accordance with the local legislation and institutional requirements. Written informed consent from the participants was not required to participate in this study in accordance with the national legislation and the institutional requirements.

## Author Contributions

Y-QG, JT, D-YW, and T-SZ: conception and design. Y-QG, M-LW, JM, JG, KL, J-JW, and T-SZ: provision of study materials or patients. Y-QG, JT, M-LW, JG, KL, and J-JW: collection and assembly of data. Y-QG, JT, M-LW, JG, and D-YW: data analysis and interpretation. Y-QG, JT, M-LW, JG, D-YW, and T-SZ: manuscript writing. All authors: final approval of manuscript.

## Conflict of Interest

The authors declare that the research was conducted in the absence of any commercial or financial relationships that could be construed as a potential conflict of interest.
